# A Multicenter Retrospective Cohort Study Assessing the Incidence of Anemia in Patients Associated With Uterine Fibroids

**DOI:** 10.7759/cureus.69899

**Published:** 2024-09-22

**Authors:** Mohamed S Keshta, Mohannad Ghanem, Yahia Alsayed, Osama Zeidan, Yousef Khorma, Rafiea Jeddy, Ahmed S Keshta, Hosni Malas, Nawal Dayoub

**Affiliations:** 1 College of Medicine, Royal College of Surgeons in Ireland - Bahrain, Busaiteen, BHR; 2 Obstetrics and Gynecology, Royal College of Surgeons in Ireland - Bahrain, Busaiteen, BHR; 3 Orthopedics, Royal College of Surgeons in Ireland - Bahrain, Busaiteen, BHR; 4 Obstetrics and Gynecology, King Hamad University Hospital, Muharraq, BHR; 5 Obstetrics and Gynecology, Assisted Reproduction in Gynecology Center, London, GBR

**Keywords:** anemias, fibroids, hysterectomy, menorrhagia, myomectomy

## Abstract

Objectives: Uterine fibroids are benign tumors that develop from the smooth muscle tissue of the uterus, typically during a woman's reproductive years. A substantial proportion of women with uterine fibroids exhibit symptoms, including menorrhagia that considerably compromise their quality of life. This study aims to characterize the fibroid subtype most commonly associated with the incidence of anemia.

Methods: This retrospective multi-center cohort study investigated the incidence of anemia in premenopausal women who were diagnosed with uterine fibroids between January 2016 and December 2022. Fibroid position, size, location, and number were assessed by trans-abdominal/vaginal ultrasound and hysteroscopy and correlated to the pre-treatment hemoglobin level.

Results: Three-quarters of patients (n=6) with submucosal fibroid presented with any form of anemia followed by 59.3% (n=115) of patients with intramural fibroid and only 36.8% (n=25) of patients with sub serousal fibroid. Interestingly, there was no difference between the groups with regard to the severity of anemia at presentation. Most red blood cells (RBCs) and hemoglobin (Hb) indices were comparable between the groups, except for mean corpuscle volume (MCV) as it was significantly lower in patients with intramural fibroids. Moreover, our investigations showed that the submucosal fibroids tend to present in a higher number as well as the biggest in size. The multivariable logistic regression showed that subserosal fibroids are associated with the lowest risk of developing anemia.

Conclusion: In conclusion, our results suggest that the occurrence of anemia should always be considered in women with submucosal fibroids. However, after adjusting for other contributing factors such as menorrhagia, submucosal fibroid did not show an increase in the risk of anemia.

## Introduction

According to the American College of Obstetrics and Gynecology, uterine fibroids are benign tumors that arise from the smooth muscle of the human uterus during the childbearing age. They are clinically major causes of morbidity affecting up to 25% of women of which 50% of them are symptomatic with substantial negative changes in life [1,2]. Nonetheless, the clinical features of fibroid presentation are variable, they consistently correlate with their location, number, and size. Symptoms associated with uterine fibroids include menorrhagia, dysmenorrhea, infertility, and pregnancy loss [3]. In premenopausal women, menorrhagia is presumably the most prominent symptom caused by fibroid which is known to negatively affect the serum iron level of childbearing women. In the absence of appropriate management, the serum hemoglobin may gradually decrease [4]. Excessive menorrhagia results in iron deficiency anemia which is considered a serious health issue and increases the need for hysterectomy in those who prefer to preserve their fertility or uterus [5]. Moreover, bleeding can also be associated with fibroid in postmenopausal women but bleeding in this population should be evaluated for worrisome causes including endometrial hyperplasia and carcinoma [6]. Anemia is associated with clinical features including fatigue, epithelial changes, oral lesions, dysphagia, and reduced immune response [7]. Furthermore, acute ischemic stroke, venous stasis, and retinopathy are being considered as consequences of severe anemia [8,9]. Biologically, anemia occurs due to decreased erythrocyte production because of impaired proliferation of red cell precursors or ineffective maturation of erythrocytes or increased loss of erythrocytes due to hemolysis or blood loss [10]. Hemoglobin concentration is the most common diagnostic test for assessing anemia [11].

Ultrasound is the standard diagnostic tool for characterizing the presence and growth of uterine fibroids. However, pelvic MRI provides the most accurate features in terms of the number, size, and location of all fibroids in the uterus. The use of these imaging modalities assisted in classifying the fibroids into subgroups including subserosal, submucosal, and intramural according to their location in the uterus. Clinically, any fibroid in the uterus is often considered responsible for heavy menstrual bleeding (HMB) even if they do not affect the uterine cavity [12]. Submucosal fibroids are thought to be more likely to cause anemia since it is located at the uterine lining, hence increasing the lining area which results in HMB. Several studies supported this finding by demonstrating an improvement in HMB in patients who had undergone hysteroscopic myomectomy [13,14]. On the other hand, additional studies have contradicted this concept due to the lack of association between HMB and submucosal fibroid. An ultrasound-based cross-sectional study has illustrated a similar amount of bleeding in both submucosal fibroid and non-submucosal fibroid [15]. Moreover, intramural fibroids have been considered a cause of HMB due to their location in the uterine muscle which results in an increase in the size of the uterus cavity [16]. However, a sonohysterography-based study found that both intramural fibroids and submucosal fibroids were associated with equal amount of bleeding [17].

This study includes a large database from the principal healthcare providers which makes it well-suited to determine the correlation of anemia incidence in different types of fibroids. Early classification of fibroid types may contribute to a reduction of the incidence of severe anemia, thereby reducing both the clinical and economic burden associated with hysterectomy.

## Materials and methods

**Figure 1:**
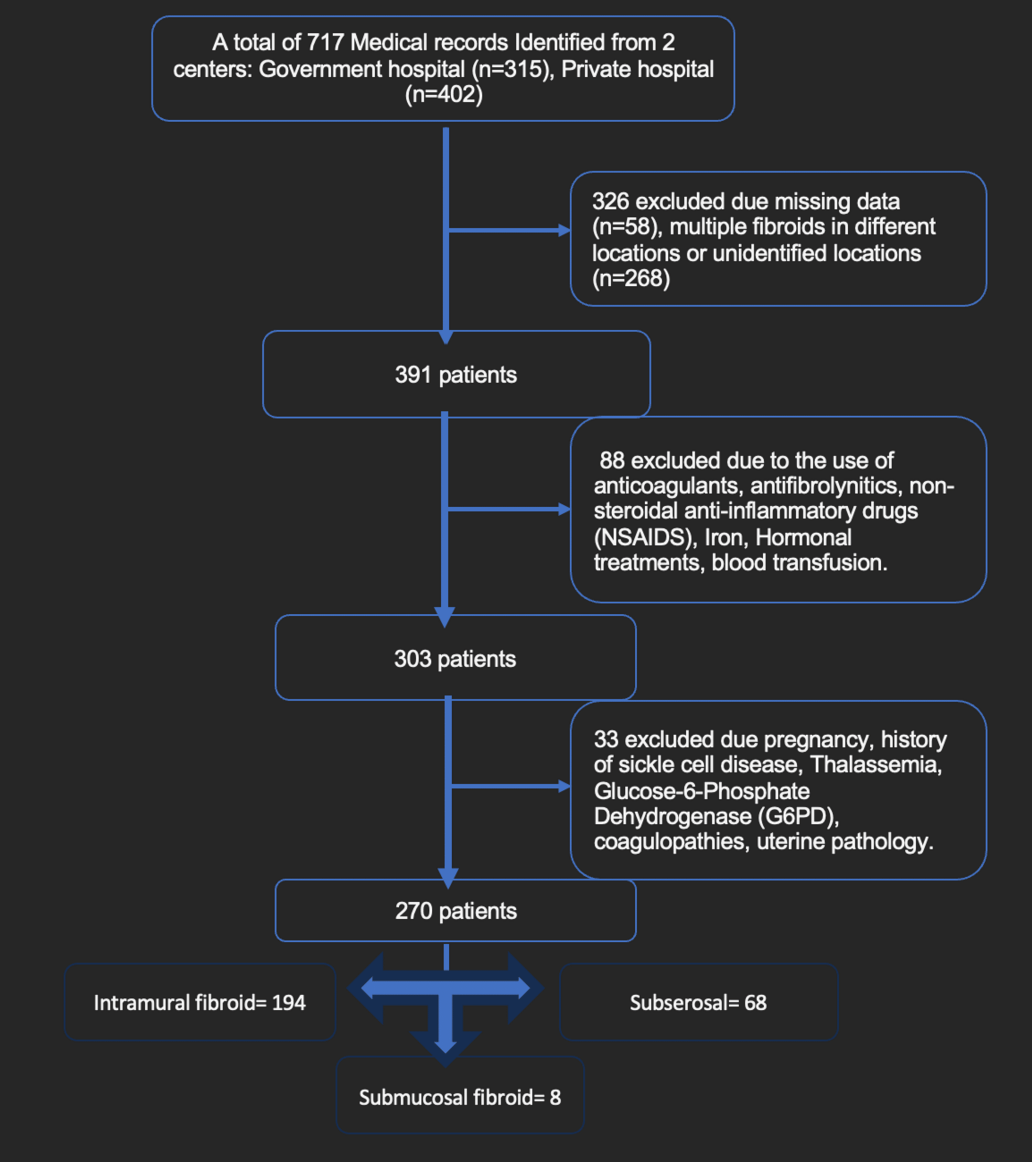
Flow chart on data collection including the inclusion and exclusion criteria

This retrospective multi-center cohort study investigated the incidence of anemia in premenopausal women who were diagnosed with uterine fibroids between January 2016 and December 2022. This data was collected at the Gynecology clinic in two hospitals (Government and Private) across the Kingdom of Bahrain including King Hamad University Hospital (KHUH) and American Mission Hospital (AMH). The study design was approved by the ethical committee at the research center in all participating hospitals. All data included were anonymized. Computerized medical records were reviewed with confidentiality. Demographics, clinical data, and laboratory variables were abstracted from the patient’s electronic medical records. Exclusion criteria were menopause, pregnancy, multiple fibroids in different locations, uterine pathologic conditions (adenomyosis, endometrial polyp, intrauterine adhesions, endometrial hyperplasia, endometrial cancer), use of anticoagulants, use of antifibrinolytic agents, use of non-steroidal anti-inflammatory drugs (NSAIDs), coagulopathies (hemophilia, von Willebrand disease, leukemia, thrombocytopenia, vitamin K deficiency, hepatic failure, disseminated intravascular coagulation, and autoimmune disease), use of hormones (oral contraceptives, gonadotropin-releasing hormone (GnRH) agonists, intrauterine system), body mass index (BMI) <19, iron supplement or blood transfusion within three months. Furthermore, patients taking iron therapy were automatically excluded. After applying our exclusion criteria, the total number of patients included is 270 patients (Figure 1).

Patients who presented with menorrhagia were asked to quantify the number of tampons and pads used per day for each day in their cycle. Hemoglobin, mean corpuscle volume (MCV), and ferritin were determined at the first visit before any use of diagnostic tools or medical treatment, e.g., blood transfusion or iron supplements. Anemia severity was classified into three groups: mild (10.1-12 g/dL), moderate (8.1-10 g/dL), and severe (≤ 8 g/dL) [18]. All the official radiological reports were reviewed. The ultrasound sonography (USS) was done at the gynecology clinics to assess fibroids’ number, position, location, and size. USSs were carried out trans-abdominally and trans-vaginally. MRI post-contrast dynamic study was carried out in axial, sagittal, and coronal planes.

Fibroids were classified based on tissue-layer location according to the International Federation of Gynecology and Obstetric (FIGO) into the three major types of uterine fibroid: intramural, submucosal, and subserosal. Intramural fibroid refers to the tumor that grows within the myometrium and can be in contact with the endometrium with no extension to the endometrial cavity. Submucosal fibroid refers to the tumor that extends into the endometrium underneath the mucosal lining. Subserosal fibroid refers to the tumor that is in the outer contour of the uterus beneath the uterine peritoneum [12,19].

Statistical analysis was done using StatsDirect software (version 3.3.5 22/03/2021; StatsDirect Ltd., Birkenhead, United Kingdom). The first assessment was done on the data to explore differences between the groups with regard to basic characteristics such as age, BMI, parity, and smoking. Furthermore, symptoms at presentation and occurrence of anemia were compared between the groups. One-way analysis of variance (ANOVA) was used for normally distributed continuous variables, the Kruskal-Wallis test was used for non-parametric variables, and Chi-square or Fisher tests were used for categorical variables. Multiple logistic regression analyses were performed to identify the effect of the type of fibroids on the risk of diagnosing anemia, after adjusting results for age, BMI, parity, smoking, size of the fibroid, and menorrhagia as covariates. Results were presented as odds ratios (OR) with 95% confidence interval (CI) and P-values. P-values of less than 0.05 were considered statistically significant.

## Results

Patient demographics and distribution of fibroid types

A total of 270 patients were included in this study. Of them, 194 patients had intramural fibroid, eight patients had submucosal fibroid, and 68 patients had subserosal fibroid. Table 1 shows the characteristics of the study population based on type of hospital presentation, previous pregnancy, previous parity, body mass index, age, nationality, and smoking. More patients with submucosal fibroids were seen in government hospitals at 62.5% compared to 37.9% of intramural fibroids and 25% of subserosal fibroids (p=0.04). The groups were matched with regard to previous pregnancy/parity, age, nationality, and smoking. Patients with BMI >30 and ≤35 subgroups were significantly likely to be presented with submucosal fibroids at 57.1% compared to 19.3% of intramural and 31.7% of subserosal fibroids (p=0.03).

**Table 1:** Medical centers and characteristics of the study population Numbers are shown with percentages in parentheses (n (%)).

Category	Intramural Fibroid (N=194)	Submucosal Fibroid (N=8)	Subserosal Fibroid (N=68)	P-value
Hospital				
Private	121 (62.1)	3 (37.5)	51 (75)	0.04
Government	74 (37.9)	5 (62.5)	17 (25)	
Previous pregnancy (mean ± SD)	1.9 ± 1.8	1± 1.4	1.5±1.6	0.41
Previous parity (mean ± SD)	1.7± 1.5	0.8± 1.4	1.5±1.6	0.29
BMI				
18 to ≤25	36 (20.5)	3 (42.9)	17 (28.3)	0.26
>25 to ≤30	70 (39.8)	0 (0)	18 (30)	0.05
>30 to ≤35	34 (19.3)	4 (57.1)	19 (31.7)	0.03
>35 to ≤40	26 (14.8)	0 (0)	4 (6.7)	0.14
>40	10 (5.7)	0 (0)	2 (3.3)	0.64
Age (mean ± SD)	43.7± 7.9	38.4± 4.8	41.3±10.4	0.36
Bahraini/Saudi	94 (48.7)	5 (62.5)	33 (48.5)	0.69
Indian/Pakistani	42 (21.8)	2 (25)	13 (19.1)	0.87
Others	57 (29.5)	1 (12.5)	22 (32.4)	0.5
Smoking	7 (3.6)	0 (0)	3 (4.4)	0.79

Symptom comparison and clinical findings among fibroid types

There was no significant difference between the groups with regard to most symptoms associated with fibroids including menorrhagia and pelvic pain. It is noteworthy that dysmenorrhea was found highly prevalent among women with submucosal fibroid at 37.5% compared to 26.2% in patients with intramural fibroids and 13.2% in patients with subserosal fibroid (p=0.03). The groups had similar blood pressure and heart rate assessments at presentation (Table 2).

**Table 2:** Signs and symptoms of anemia and fibroid at the time of presentation Numbers are shown with percentages in parentheses (n (%)).

Symptom/Characteristic	Intramural Fibroid (N=194)	Submucosal Fibroid (N=8)	Subserosal Fibroid (N=68)	P-value
Pelvic pain	113 (57.9)	5 (62.5)	35 (51.5)	0.68
Frequent urination	8 (4.1)	0 (0)	5 (7.4)	0.66
Constipation	2 (1.03)	0 (0)	2 (2.9)	0.36
Backache	80 (41)	2 (25)	22 (32.4)	0.38
Irregular period	57 (29)	3 (37.5)	17 (25)	0.63
Menorrhagia	117 (60)	5 (62.5)	31 (45.6)	0.11
Dysmenorrhea	51 (26.2)	3 (37.5)	9 (13.2)	0.04
Dyspareunia	6 (3.1)	0 (0)	1 (1.5)	0.74
Post-coital bleeding	0(0)	0 (0)	2 (2.9)	0.12
Amenorrhea	2 (1.03)	0 (0)	3 (4.4)	0.24
Intermenstrual bleeding	6 (3.1)	0 (0)	2 (2.9)	>0.99
Fatigue	34 (17.4)	2 (25)	16 (23.5)	0.45
Pale or yellowish skin	1 (0.5)	1 (12.5)	1 (1.5)	0.06
Palpitation	4 (2.1)	0 (0)	2 (2.9)	0.71
Abdominal pain	37 (18.9)	3 (37.5)	17 (25)	0.22
Shortness of breath	2 (1.03)	0 (0)	0 (0)	>0.99
Dizziness	20 (10.3)	2 (25)	11 (16.2)	0.16
Headaches	25 (12.8)	2 (25)	12 (17.7)	0.28
Weight gain	2 (1.03)	0 (0)	1 (1.5)	>0.99
Heart rate (mean ± SD)	83.6 ± 9.7	86.3 ± 14.6	83.3 ± 10.1	0.73
Heart group	12 (6.2)	1 (12.5)	6 (8.8)	0.34
Blood pressure (BP) group	114 (58.5)	4 (50)	37 (54.4)	0.75
Systolic BP (mean ± SD)	125.6 ± 16.8	118 ± 14.1	123.9 ± 15.3	0.36
Diastolic BP (mean ± SD)	75.4 ± 10.4	74.5 ± 10.6	74.9 ± 10.6	0.94

Anemia prevalence and hematologic findings among fibroid types

The total number of patients presented with any form of anemia was 146 with a rate of 54.07%. Interestingly, there was no difference between the groups with regard to the severity of anemia at presentation. Three-quarters of patients with submucosal fibroid presented with any form of anemia followed by 59.3% of patients with intramural fibroid and only 36.8% of patients with subserousal fibroid (p=0.003). This presentation reflected on similar differences in the level of Hb values (p=0.02) and hematocrit values (p=0.03) in the relevant groups. On the further assessment of RBC and Hb indices, most of the parameters were comparable between the groups except MCV as it was significantly lower in patients with intramural fibroids at 79.7% followed by patients with submucosal fibroids at 81.5% compared to 82.9% in the patients with the subserosal fibroid (p=0.03) (Table 3).

**Table 3:** Patients’ laboratory results at the time of presentation Numbers are shown with percentages in parentheses (n (%)). Hb: hemoglobin; RBC: red blood cells; WBC: white blood cells; Hct: hematocrit; MCV: mean corpuscular volume; MCH: mean corpuscular hemoglobin; MCHC: mean corpuscular hemoglobin concentration; RDW: red cell distribution width

Parameters	Intramural Fibroid (N=194)	Submucosal Fibroid (N=8)	Subserosal Fibroid (N=68)	P-value
Hb (g/dL) (mean ± SD)	11.6 ± 1.7	11.5 ± 1.4	12.2 ± 1.4	0.02
Anemia (Hb <12 g/dL)	115 (59.3)	6 (75)	25 (36.8)	0.003
Mild anemia (10.1-12 g/dL)	84 (43.3)	5 (62.5)	21 (30.9)	0.09
Moderate anemia (8.1-10 g/dL)	26 (13.4)	1 (12.5)	3 (4.4)	0.13
Severe anemia (≤8 g/dL)	5 (2.6)	0 (0)	1 (1.5)	0.79
Platelets (×10^9/L) (mean ± SD)	300.3 ± 81.5	303.1 ± 64.1	276.9 ± 74.9	0.38
WBC (×10^9/L) (mean ± SD)	7.5 ± 2.8	7.3 ± 1.7	7.2 ± 2.5	0.69
RBC (×10^12/L) (mean ± SD)	4.5 ± 0.6	4.5 ± 1.1	4.5 ± 0.5	0.95
Hct (%) (mean ± SD)	35.8 ± 4.1	34.8 ± 3.5	37.3 ± 4.5	0.03
MCV (fL) (mean ± SD)	79.7 ± 8.5	81.5 ± 7.3	82.9 ± 6.8	0.03
MCH (pg) (mean ± SD)	26.1 ± 3.9	25.9 ± 4.2	26.9 ± 2.9	0.25
MCHC (g/dL) (mean ± SD)	32.1 ± 2.3	32.1 ± 3.4	32.8 ± 1.7	0.08
RDW (%) (mean ± SD)	14.6 ± 2.7	14.7 ± 2.4	14.1 ± 2.3	0.35

Comparison of imaging modalities and management approaches for submucosal, intramural, and subserosal fibroids

Moreover, our investigations showed that the submucosal fibroids tend to present in a higher number (more than four fibroids per woman) as well as the biggest in size >50mm (p=0.01 and p=0.03, respectively). Fibroids sized between ≥20 mm and ≤50 mm were seen in 50% of patients with intramural fibroids compared to 35.5% in patients with subserosal fibroids and 12.5% in patients with submucosal fibroids (p=0.02). Reasonably, our analysis revealed that ultrasound was the most common imaging tool in detecting intramural and subserosal fibroids (p=0.005). On the contrary, MRI was the usual tool of choice in the diagnosis of submucosal fibroid (p=0.04). Intramural fibroids were likely to be investigated using the abdominal scan in 81.1% of patients compared to 75% of submucosal fibroids and 63.5% of subserosal fibroids (p=0.01). There was no difference between the groups with regard to management approach; however, only 25% of patients with subserosal fibroids received iron treatment compared to 45.6% of patients with intramural fibroids and 50% of patients with submucosal fibroids (p=0.009) (Table 4).

**Table 4:** Fibroid data including ultrasound and MRI diagnosed fibroids and management approaches Numbers are shown with percentages in parentheses (n (%)).

	Intramural Fibroid (N=194)	Submucosal Fibroid (N=8)	Subserosal Fibroid (N=68)	P-value
Fibroid number				
1	115 (58.9)	4 (50)	45 (66.2)	0.49
2	43 (22.1)	0 (0)	10 (14.7)	0.15
3	15 (7.7)	1 (12.5)	10 (14.7)	0.24
≥4	22 (11.3)	3 (37.5)	3 (4.4)	0.01
Size				
<20 mm	24 (12.4)	1 (12.5)	10 (14.7)	0.88
≥20 to ≤50 mm	97 (50)	1 (12.5)	24 (35.3)	0.02
>50 mm	73 (37.6)	6 (75)	34 (50)	0.03
Diagnostic tool				
Ultrasound	173 (89.6)	4 (50)	58 (85.3)	0.005
Hysteroscopy	2 (1)	0 (0)	0 (0)	0.67
MRI	9 (4.7)	2 (25)	3 (4.4)	0.04
Multiple	9 (4.7)	2 (25)	7 (10.3)	0.03
Ultrasound				
Abdominal	142 (81.1)	3 (75)	40 (63.5)	0.01
Vaginal	33 (18.9)	1 (25)	23 (36.5)	
Surgical treatment				
Myomectomy	33 (17.1)	2 (25)	18 (26.9)	0.22
hysterectomy	33 (17.1)	2 (25)	7 (10.5)	0.32
None	127 (65.8)	4 (50)	42 (62.6)	0.6
Hormonal admission post-diagnosis	50 (25.6)	1 (12.5)	10 (14.7)	0.13
Iron admission post-diagnosis	89 (45.6)	4 (50)	17 (25)	0.009

Impact of fibroid type on anemia risk: Insights from multivariable logistic regression

Multivariable logistic regression was performed to assess the effect of different types of fibroids on the risk of developing anemia. After adjusting all other contributing risk factors such as the patient's age, size of the fibroid, and associated symptoms such as menorrhagia or intermenstrual bleeding (IMB). Patients presented with subserosal fibroids have a reduced risk of developing anemia with OR of 0.41 (0.23-0.73) for any form of anemia (p=0.002), OR=0.46 (0.26-0.79) for mild anemia (p=0.006), and OR=0.26 (0.09-0.72) for moderate anemia. Furthermore, there was no notable increase in the risk of anemia in submucosal and intramural fibroids (p=0.009) (Table 5).

**Table 5:** Multivariable logistic regression analysis among fibroid types associated with anemia OR: odds ratio; CI: confidence interval

	Intramural Fibroid (N=194)	Submucosal Fibroid (N=8)	Subserosal Fibroid (N=68)
Anemia OR (95% CI)	0.9 (0.54-1.49), P = 0.69	1.95 (0.55-6.93), P = 0.29	0.41 (0.23-0.73), P = 0.002
Mild anemia OR (95% CI)	0.7 (0.44-1.12), P = 0.14	1.6 (0.52-4.95), P = 0.42	0.46 (0.26-0.79), P = 0.006
Moderate anemia OR (95% CI)	0.81 (0.4-1.65), P = 0.57	0.7 (0.13-3.63), P = 0.67	0.26 (0.09-0.72), P = 0.009

## Discussion

This study identified changes to serum Hb and MCV in relation to the type of fibroid. We compared the data of three different types of fibroids: intramural, submucosal, and subserosal fibroids. As presented in Figure 1, the prevalence of intramural fibroids, submucosal, and subserosal fibroids represent 72%, 3%, and 25% of the studied population, respectively. The prevalence of fibroids varies among different studies based on their aim and methodology. In fact, a PubMed search using the term “uterine fibroids” provides over 15000 results over the past 20 years. Additionally, a search using the terms “uterine fibroids” AND “anaemia” provides 336 results. Eventually, adding the term “Epidemiology” or “Prevalence” provides only 50-60 results over the same period. Therefore, a comparative discussion using the available literature is prone to selection and/or reporting bias. Finally, it was revealed that fibroid location, BMI, fibroid number, and fibroid size are significant risk factors that determine serum Hb levels for women with uterine fibroids.

This study revealed that submucosal fibroids were significantly larger in diameter than intramural and subserosal fibroids; consequently, they were the most associated with low serum Hb levels. A retrospective cohort study of 3,500 women by Puri et al. established a strong association between submucosal fibroids and low Hb with increased risk of anemia, particularly in classes 0 and 1 fibroid [20]. A more recent study by Ricci et al. outlined an 81% increased risk of anemia and low Hb in type 0 myoma (submucosal fibroid class 0) regardless of the presence of menorrhagia or self-reported symptoms, and an increased risk of anemia with types 1 and 2 in the presence of menorrhagia or if myomas are >60 mm [5]. Contrary to these findings by Ricci et al., our study found no association between anemia and submucosal fibroid in the absence of menorrhagia (p=0.11). While in fact menorrhagia was insignificant, dysmenorrhea, on the other hand, was noted in high prevalence among women with submucosal fibroids (37.5%) compared to intramural fibroids (26.2%) and serosal fibroids (13.2%) (p=0.04). This further aligns with an old cross-sectional cohort in 2004 showing the disassociation between uterine leiomyomas and menstrual cycle characteristics (i.e., cycle and flow lengths and heaviness of flow) [21].

On the contrary, a case series consisting of 357 patients showed that fibroid patients have equal tendencies to report heavy bleeding or be diagnosed with anemia regardless of the location of the fibroid; however, fibroid size was not accounted for [22]. Nonetheless, this study exhibited significant anemia in submucosal fibroids that were more prominent in larger fibroids. A study by Yang et al. measured serum Hb in relation to fibroid diameter and protruding proportion into the uterine cavity. The results yielded a strong negative correlation with serum Hb in regard to the submucosal fibroid diameter and protrusion, further aligning with the findings of the study. Nevertheless, Yang et al.'s study also found that myomas <2 cm had similar serum Hb regardless of their protruding proportion [23].

After controlling other factors, our findings demonstrated no increased risk of anemia in the submucosal fibroid. On the other hand, Ricci et al. noted the only significant risk factors for anemia were the grade of myoma protrusion and menorrhagia; however, our study revealed no significant correlation between anemia and menorrhagia. Thus, the only attributable risk of anemia is a degree of fibroid protrusion. This could support the early diagnosis of mild to moderate anemia in submucosal fibroid patients based on the characteristics of myoma. In addition, this could contribute to an objective criterion defining the management plan of types 0 and 1 myomas in comparison to types 2 and 3 (based on the tissue layer of the myoma and the degree of protrusion into the uterine cavity). Currently, there are no definitive criteria for the medical management of fibroids due to their limited efficacy and mainly revolve around symptomatic relief [24].

A noteworthy addition is that submucosal fibroids can cause heavy vaginal bleeding in several ways. Submucosal fibroids are frequently associated with endometritis, which enhances the presence of hypermenorrhea. Moreover, the presence of endometrial lining ulceration increasingly led to IMB [25-27]. These facts can potentially explain our study findings of more patients with submucosal fibroids being presented in government hospitals as they might require early referral and urgent management at the hospital.

An additional finding of this study demonstrated the presence of low MCV in intramural fibroid patients. Intramural fibroids, unexpectedly, exhibited the lowest MCV compared to submucosal and subserosal fibroids (p=0.03). This outcome contradicts the other findings exhibiting no change in bleeding tendencies across the different types of fibroids. Furthermore, studies show microcytic anemia as the result of abnormal uterine bleeding or menorrhagia both of which are not particular features associated with intramural fibroids in this study [28,29].

The prevalence of fibroids varies among different studies based on the type of investigation and method of diagnosis. Of 270 patients with fibroids, MRI showed a higher sensitivity to detect submucosal fibroids compared to intramural or subserosal fibroids. Intramural and subserosal fibroids were commonly detected by US compared to submucosal. On the contrary, a study was conducted on 121 surgically confirmed fibroids by Levens et al. aimed to measure the detection rate of different types of fibroids using different diagnostic modalities. The study showed that MRI was associated with a higher detection rate of intramural and subserosal compared to submucosal fibroids. Furthermore, US has missed a higher number of intramural and subserosal fibroids compared to the missed diagnosis of submucosal [30]. Previous studies in the literature suggest that MRI is the best diagnostic tool in detecting uterine fibroids and it has superior sensitivity to detect small fibroids [31,32].

Logistic regression of our data revealed that intramural and submucosal fibroids are associated with an unnoticeable risk of anemia, subserosal fibroid was correlated with a reduced risk of anemia. A retrospective study by Sulaiman et al. aimed to investigate the effect of fibroid size on menstrual blood loss and found an inverse relationship between fibroid diameter and risk of anemia [33].

Some limitations of this study can be attributed to the small size of the population, which included a total number of 270 patients with unequal distribution among different types of fibroids. There were also no unified criteria for quantifying menorrhagia or other symptoms; data was rather subjective per individual and was collected retrospectively. The heterogenicity presented with patient data, due to their subjective criteria in viewing symptoms, may limit the study from accurately determining associated symptoms with each type of fibroid. Lastly, even though the number of patients with submucosal fibroids had the least prevalence in this study, it had the greatest impact on anemia which can be secondary to sample bias. One approach to tackle these inaccuracies is increasing the size of the population. Nonetheless, the ability of this study to yield significant changes, despite the heterogenicity, sets a baseline framework that is fixed and may be used as a tool to establish a definitive approach to treating and monitoring the severity or progression of uterine fibroids.

## Conclusions

In conclusion, this study highlights that submucosal fibroids, especially those with larger diameters, are more closely associated with anemia due to low serum Hb levels. While previous studies, such as those by Ricci et al., found a strong correlation between submucosal fibroids and anemia, particularly in cases with menorrhagia, our findings suggest that anemia is more related to the degree of fibroid protrusion into the uterine cavity than the presence of menorrhagia. Furthermore, while submucosal fibroids are often associated with heavy vaginal bleeding and endometrial complications, our study also revealed that intramural fibroids may unexpectedly contribute to lower MCV, despite being less commonly linked to bleeding.

The study also emphasizes the importance of early diagnosis and management of fibroids based on the specific characteristics of the fibroid, particularly submucosal types. However, the small sample size and subjective symptom reporting limit the study’s accuracy, and future research with a larger, more homogenous population is needed to further refine diagnostic and treatment approaches for uterine fibroids. Despite these limitations, the findings provide valuable insights that could inform better management strategies for fibroid-associated anemia.
